# Human Meningiomas Reveal No Evidence of Neuroendocrine Differentiation

**DOI:** 10.1111/apm.70177

**Published:** 2026-03-02

**Authors:** Sofie Eline Tollefsen, Anders Hagen Jarmund, Ole Solheim, Ida Kaalhus Nordahl, Thi My Linh Hoang, Anette H. Skjervold, Patricia Mjønes, Sverre Helge Torp

**Affiliations:** ^1^ Department of Clinical and Molecular Medicine, Faculty of Medicine and Health Sciences Norwegian University of Science and Technology (NTNU) Trondheim Norway; ^2^ Department of Neurosurgery, St. Olavs Hospital Trondheim University Hospital Trondheim Norway; ^3^ Department of Neuromedicine and Movement Science, Faculty of Medicine and Health Sciences Norwegian University of Science and Technology (NTNU) Trondheim Norway; ^4^ Department of Biomedical Laboratory Science, Faculty of Natural Sciences Norwegian University of Science and Technology (NTNU) Trondheim Norway; ^5^ Department of Pathology, St. Olavs Hospital Trondheim University Hospital Trondheim Norway

**Keywords:** digital pathology, immunohistochemistry, MEN1, meningioma, neuroendocrine

## Abstract

Meningiomas are heterogeneous tumors and studies suggest that meningiomas might be part of MEN1 syndrome. The tumors express somatostatin receptors (SSTRs) comparable to that seen in neuroendocrine neoplasms. We aimed to explore neuroendocrine differentiation in meningiomas by investigating the following neuroendocrine markers: neural cell adhesion molecule (CD56/NCAM), chromogranin A, chromogranin B, chromogranin C, neuron‐specific enolase (NSE), secretagogin, and synaptophysin. Our findings were related to WHO grade, tumor subtype, and SSTR2 immunoreactivity. Tissue microarrays from 162 patients with intracranial meningioma underwent immunohistochemical analyses. Immunoreactivity was assessed with manual and digital analyses. Transmission electron microscopy (TEM) was used to detect secretory granules in one tumor specimen. NSE, CD56, and chromogranin B were detected in 91%, 44%, and 16% of meningiomas, respectively. The other neuroendocrine markers were mostly negative. NSE immunoreactivity was higher in WHO grade 2 tumors (*p* = 0.027) and differed among subtypes with highest and lowest immunoreactivity in meningothelial and fibrous subtypes, respectively. Chromogranin B (*p* = 0.006) and NSE (*p* = 0.003) were positively correlated to SSTR2 immunoreactivity. No secretory granules were detected. Manual and digital evaluation showed excellent agreement. Our study does not support the hypothesis of neuroendocrine differentiation in meningiomas, as chromogranin A and synaptophysin were mostly absent.

## 1 Introduction

Meningiomas are heterogenous tumors, as reflected by the 15 different subtypes described in the 2021 WHO classification [1]. Other tumors considered not to be of neuroendocrine origin, such as breast cancer and prostate cancer, may also have neuroendocrine differentiation in parts of the tumor tissue [2, 3]. Meningiomas and neuroendocrine neoplasms may both be associated with the tumor suppressor gene *multiple endocrine neoplasia type 1* (*MEN1*) and expression of somatostatin receptors (SSTRs) [4, 5, 6, 7, 8, 9, 10]. SSTR2 is most frequently expressed in both meningiomas and neuroendocrine neoplasms [9, 10, 11]. Some studies advise that meningiomas should be considered part of the MEN1 syndrome [7, 8]. These common features may suggest neuroendocrine differentiation in meningiomas, although SSTRs are also described in some non‐neuroendocrine neoplasms such as lymphomas and breast carcinomas [12, 13]. The similarities of SSTRs in meningiomas and neuroendocrine neoplasms have several implications, including that potential novel treatments for patients with meningioma are already used routinely for patients with neuroendocrine neoplasms. This includes systemic treatment with somatostatin analogs and peptide receptor radionuclide therapy targeting SSTR2 [14, 15, 16]. To our knowledge, no previous studies have systematically investigated neuroendocrine differentiation in meningiomas using multiple neuroendocrine markers, although the presence of some neuroendocrine proteins has been described in case reports and smaller studies [17, 18, 19, 20, 21, 22, 23, 24, 25, 26]. The most sensitive and specific neuroendocrine markers are currently chromogranin A and synaptophysin [10, 27, 28, 29, 30].

The primary aim of our study was to explore neuroendocrine differentiation by investigating immunohistochemical expression of neuroendocrine markers in a large series of human meningiomas. The markers included neural cell adhesion molecule (CD56/NCAM), chromogranin A, chromogranin B, chromogranin C, neuron‐specific enolase (NSE), secretagogin, and synaptophysin. The secondary aims were to [1] relate the immunoreactivity of neuroendocrine markers to WHO grade, tumor subtype, and SSTR2 immunoreactivity and [2] in one case examine the presence of secretory granules with transmission electron microscopy (TEM). Immunoreactivity was evaluated using two different methods: manual evaluation with conventional microscopy and our previously developed pixel analyses in ImageJ [9].

## 2 Material and Methods

### 2.1 Patients

This study included 162 adult patients operated for primary intracranial WHO grade 1 or 2 meningioma between January 1, 1991 and December 31, 2000 at St. Olavs hospital, Trondheim University Hospital, Trondheim, Norway. All specimens were later reviewed and graded according to the 2016 WHO classification by an experienced neuropathologist [31, 32]. The selection of patients and the immunoreactivity of SSTR2 have previously been reported [9, 33].

### 2.2 Immunohistochemistry

Tissue microarray (TMA) preparation was previously conducted by Arnli et al. [34]. Paraffin sections of 4 μm were heated to 60°C for 1 h preceding deparaffinization and rehydration. Heat Induced Epitope Retrieval (HIER) was utilized in Target Retrieval Solution (TRS) pH 9 for 20 min at 97°C before cooling to 65°C, using PT‐Link (Dako Denmark A/S, Glostrup, DK). Immunodetection was automated by Dako Autostainer Plus. Details on primary antibodies are described in Table S1. Dako REAL Peroxidase Blocking Solution (S2023) was applied for 10 min to prevent endogenous peroxidase activity. Sections were then incubated with secondary antibodies with horseradish peroxidase for 30 min (HRP Mouse/Rabbit EnVision—Polymer, Dako REAL Envision detection System K 5007). Prior to hematoxylin counterstain, DAB+ chromogen (Dako REAL Envision detection System) was applied for 10 min. Tumor samples and positive controls of pancreas, duodenum and thyroid were treated in the same manner. The primary antibodies were omitted for negative control. Three TMA cores were obtained from each patient, consisting of tumor tissue without hemorrhage or necrosis. All TMA cores were scanned using Olympus VS120S5 with a ×20 objective lens, and each TMA core had a diameter of 1000 μm. The total number of pixels per image was 4929 × 4929. One collective score was calculated for all three TMA cores from each patient. Only TMA cores with > 50% remaining tissue were included.

### 2.3 Scoring of Immunohistochemistry

Visually assessed staining index (SI) was defined as the product of staining intensity and percentage of positive cells, signifying that intensity was scored as negative (0), weak (1), moderate (2) or strong (3), and percentage of positive cells was recorded as < 10% (1), 10%–50% (2) or > 50% (3) [3, 9].

Pixel analyses of immunoreactivity for the seven neuroendocrine proteins were performed as previously described by Tollefsen et al. and Varghese et al. [9, 35]. The thresholds for background stain exclusion were 203 for CD56, 222 for chromogranin A, 222 for chromogranin B, 221 for chromogranin C, 220 for NSE, 221 for secretagogin and 222 for synaptophysin. The four intensity zones were: negative (intensities > 180, score 1), low positive (intensities 121–180, score 2), positive (intensities 61–120, score 3) and highly positive (intensities < 61, score 4) [35]. Digital scores (DS) were calculated as the sum of intensity zone scores weighted by the relative number of pixels in the given zone. DS were measured on a scale from 1 (only negative pixels) to 4 (only highly positive pixels). ImageJ version 1.53c was used for the image acquisition and R version 4.3.1 was used to calculate DS [9, 35].

### 2.4 Transmission Electron Microscopy (TEM)

Fresh meningioma tissue from one surgical specimen was fixed with 2.5% glutaraldehyde in 0.1 M phosphate buffer overnight, followed by a solution of 2% osmium tetroxide and 1.5% potassium ferrocyanide in the same buffer for 1 h. Both steps were carried out at room temperature. Next, the sample was dehydrated with increasing concentrations of ethanol (50%, 70%, 90% and 100%). Acetone was applied as a transitional solvent and followed by infiltration with Epon resin (25%, 50%, 75% and 100%). The tissue was then embedded in blocks and polymerized for 3 days at 60°C. Ultrathin sections were cut using an ultramicrotome (Leica EM UC7) and a diamond knife (Diatome). Counterstaining was conducted with uranyl acetate and lead citrate for electron microscopic examination with a TEM (JSM‐1011, JEOL) operating at 80 kV. Morada digital camera with Radius software (BoRAS) was used for imaging. The TEM images were reviewed by an experienced neuropathologist (S.H.T.).

### 2.5 Statistical Analyses

The correlation of SI and DS was assessed using Spearman's rank‐order correlation. Tumors with SI > 3 were considered positive. DS was used for further statistical analysis of the neuroendocrine proteins that had median DS > 1.10. Mann Whitney U test was applied for the dichotomous variable WHO grade (grade 1 versus grade 2). Associations between DS and tumor subtypes were assessed with Kruskal Wallis, and if statistically significant, followed by Dunn's test with Bonferroni correction for multiple testing. Only tumor subtypes with at least two patients were investigated: (1) meningothelial, (2) fibrous, (3) transitional and (4) atypical tumors. Spearman's rank‐order correlation was applied to investigate associations to SSTR2 immunoreactivity. A *p*‐value < 0.05 was considered significant. Statistical analyses were conducted in IBM SPSS Statistics for Windows, version 29, and R version 4.4.1.

## 3 Results

### 3.1 Patients

The study population consisted of 162 patients diagnosed with meningioma WHO grade 1 (67.3%) or WHO grade 2 (32.7%). A total of 119 (73.5%) patients were female. The median age at surgery was 60 years, ranging from 25 to 86 years. The most frequent tumor subtypes were meningothelial (17.3%), fibrous (5.6%), transitional (40.7%) and atypical (32.7%).

### 3.2 Immunohistochemical Results

**FIGURE 1:**
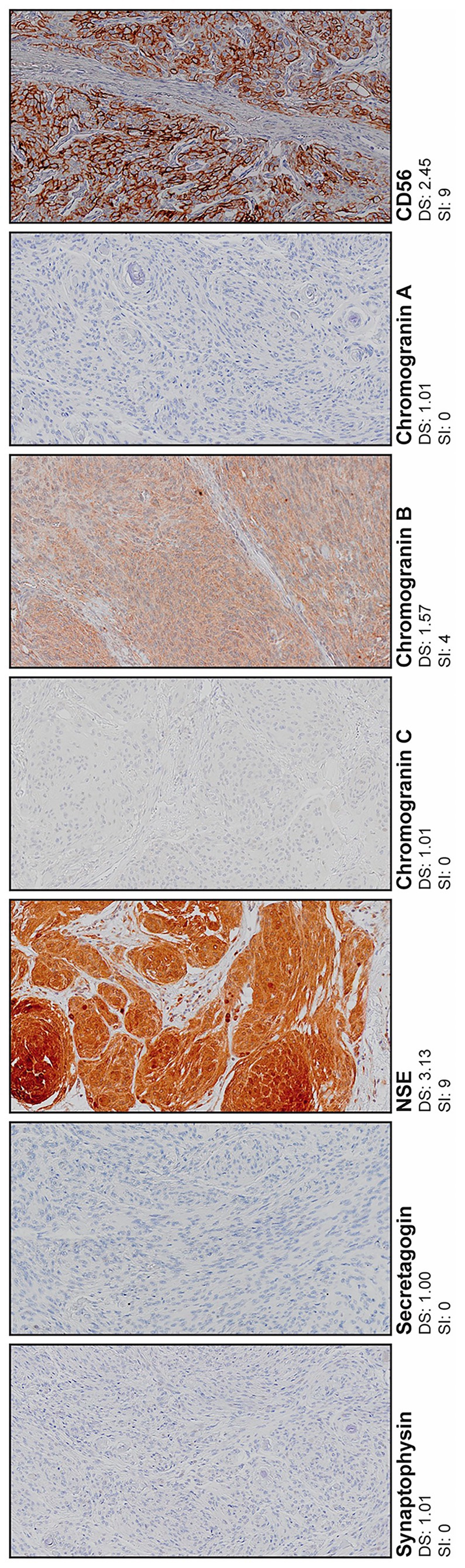
Representative tissue sections for each neuroendocrine marker. Digital scores and staining index for the presented tumor specimens are included. Original magnification, ×20. NSE, neuron‐specific enolase.

**FIGURE 2:**
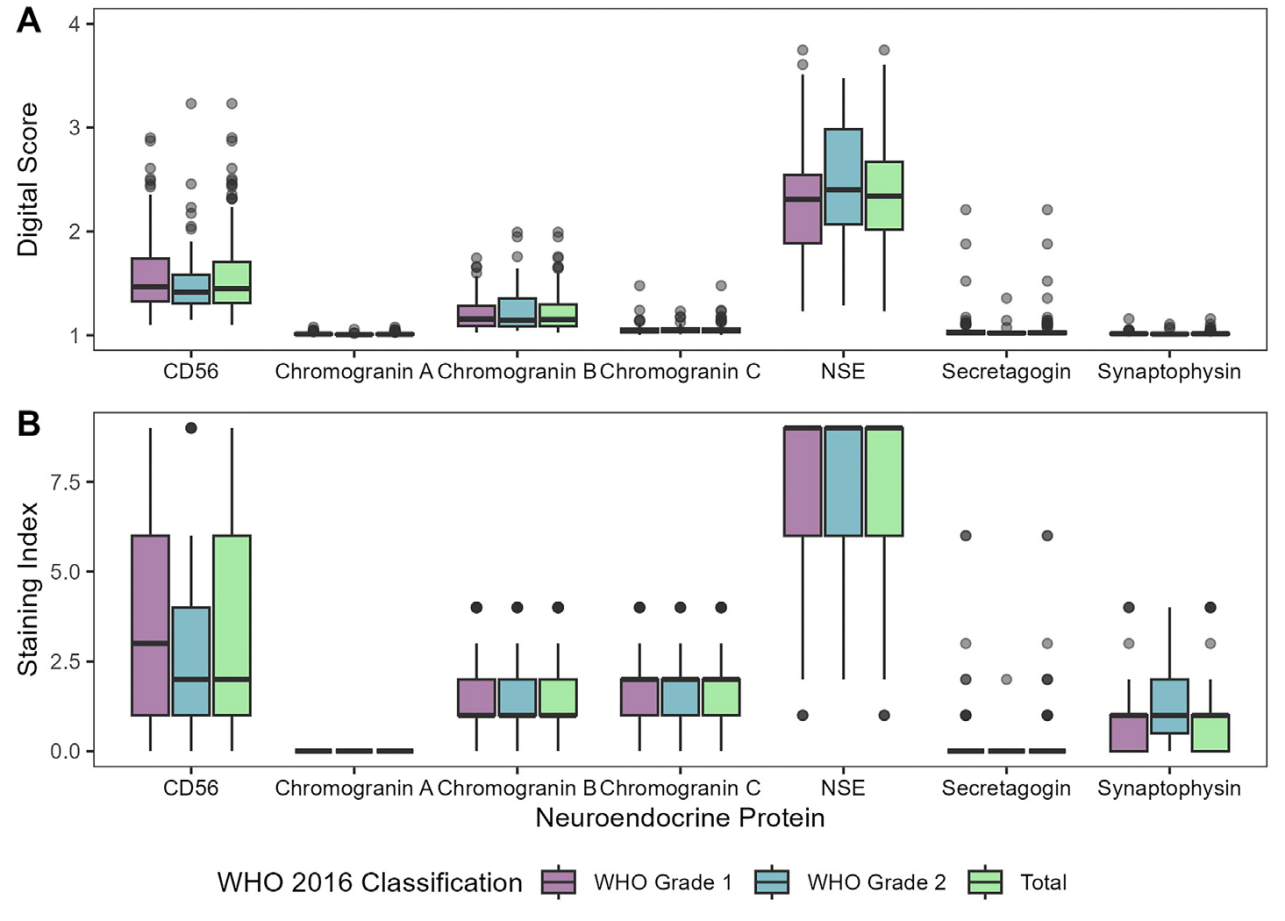
Immunohistochemical expression of neuroendocrine proteins for WHO grade 1, WHO grade 2, and collectively for all tumor cases, evaluated by (A) digital score and (B) staining index. Digital scores are calculated automatically, whereas staining index is assigned from visual inspection. Data presented as boxplot (25th percentile, median, 75th percentile). NSE, neuron‐specific enolase.

Immunoreactivity was evaluated using SI and DS. Chromogranin A had no variation in SI and no correlation was calculated. The other six proteins demonstrated significant correlations. Chromogranin A was not expressed in any tumors, while 26 (16.3%) and 12 (7.5%) tumors demonstrated cytoplasmatic immunoreactivity for chromogranin B and chromogranin C, respectively. NSE was expressed by 144 (91.1%) tumors with cytoplasmatic immunoreactivity. The 70 (44%) tumors that expressed CD56 had cytoplasmatic immunoreactivity and some membranous immunoreactivity. Two (1.2%) tumors expressed secretagogin and five (3.1%) tumors expressed synaptophysin. NSE had the highest median scores for both evaluation methods. Chromogranin A, secretagogin and synaptophysin had the lowest median scores. Fibroblasts were used as internal negative controls. Illustrations are provided in Figure 1. Results are presented as boxplots in Figure 2. All numbers are given in Tables S2–S4.

### 3.3 Relation to WHO Grade, Tumor Subtype, and SSTR2 Immunoreactivity

**FIGURE 3:**
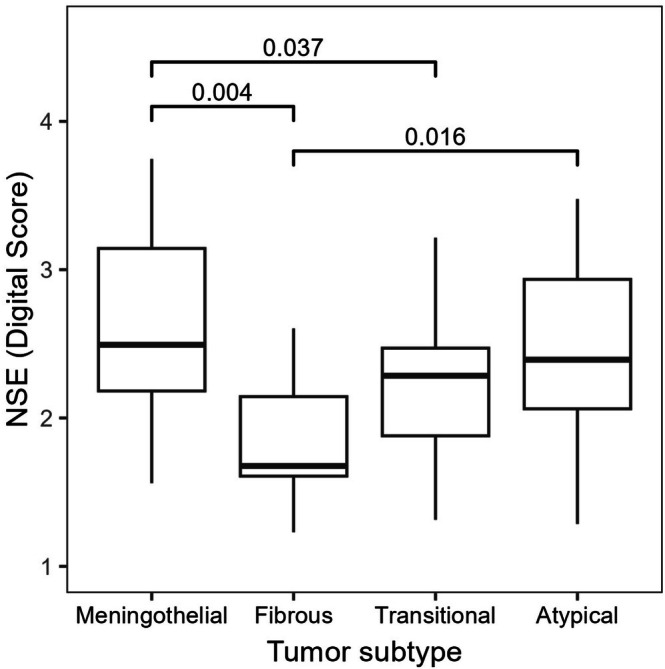
Neuron‐specific enolase (NSE) immunoreactivity related to tumor subtype according to the digital score (DS). Data presented as boxplot (25th percentile, median, 75th percentile). Significant *p‐*values are reported.

**FIGURE 4:**
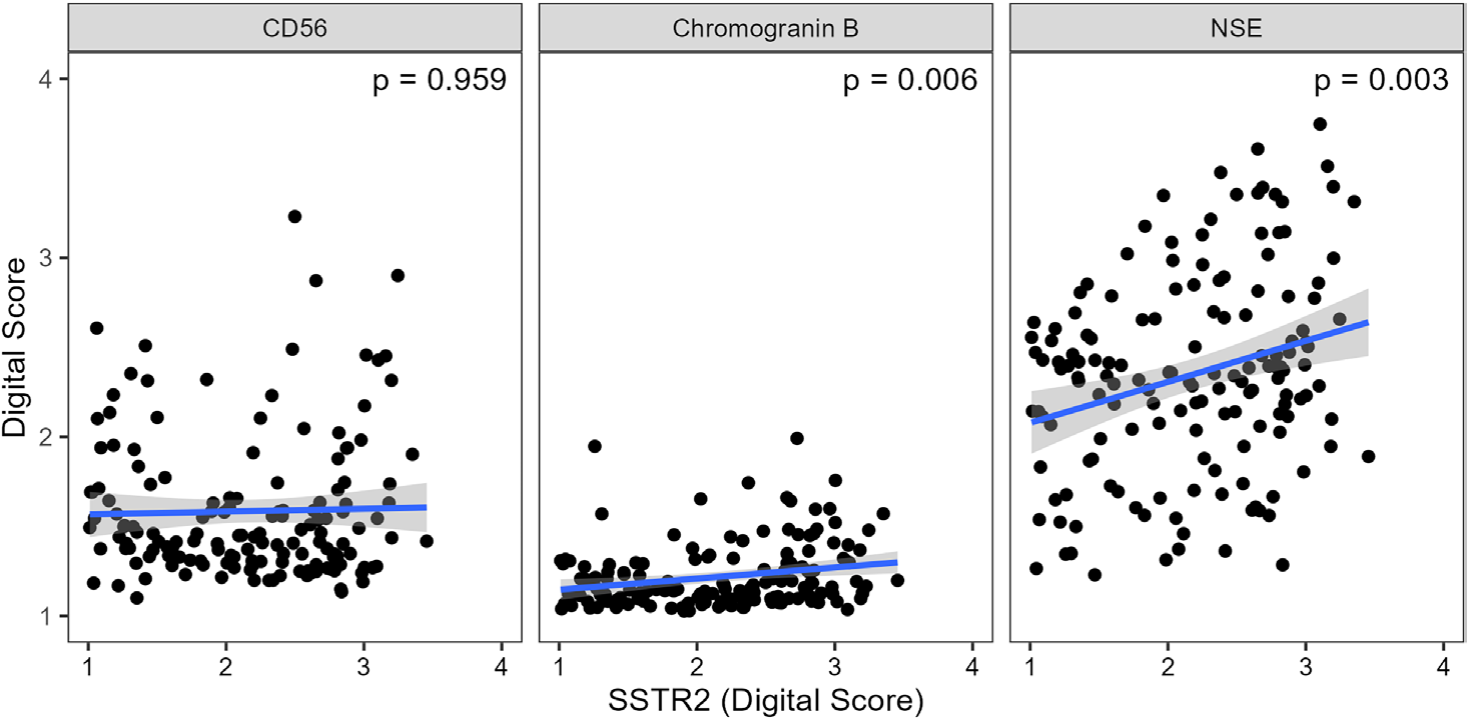
The immunoreactivity of CD56, chromogranin B, and NSE related to SSTR2 immunoreactivity. Data is presented as scatter plots with linear regression. *p*‐values are reported from Spearman's rank‐order correlations. NSE, neuron‐specific enolase.

CD56, chromogranin B and NSE had median DS > 1.10 and were tested for relations to WHO grade, tumor subtype and SSTR2 immunoreactivity. WHO grade 2 tumors had significantly higher immunoreactivity of NSE (*p‐*value = 0.027). Furthermore, NSE was less expressed in fibrous tumors compared to meningothelial subtype (adjusted *p*‐value = 0.004) and atypical tumors (adjusted *p*‐value = 0.016). Meningothelial subtypes had higher immunoreactivity than the transitional subtypes (adjusted *p*‐value = 0.037). Association to tumor subtype for NSE is illustrated in Figure 3. There was no relation to WHO grade or tumor subtype for CD56 and chromogranin B. Only the immunoreactivities of chromogranin B (*p* = 0.006) and NSE (*p* = 0.003) were significantly related to SSTR2 immunoreactivity, as illustrated in Figure 4.

### 3.4 Transmission Electron Microscopy of a WHO Grade 1 Meningothelial Meningioma

**FIGURE 5:**
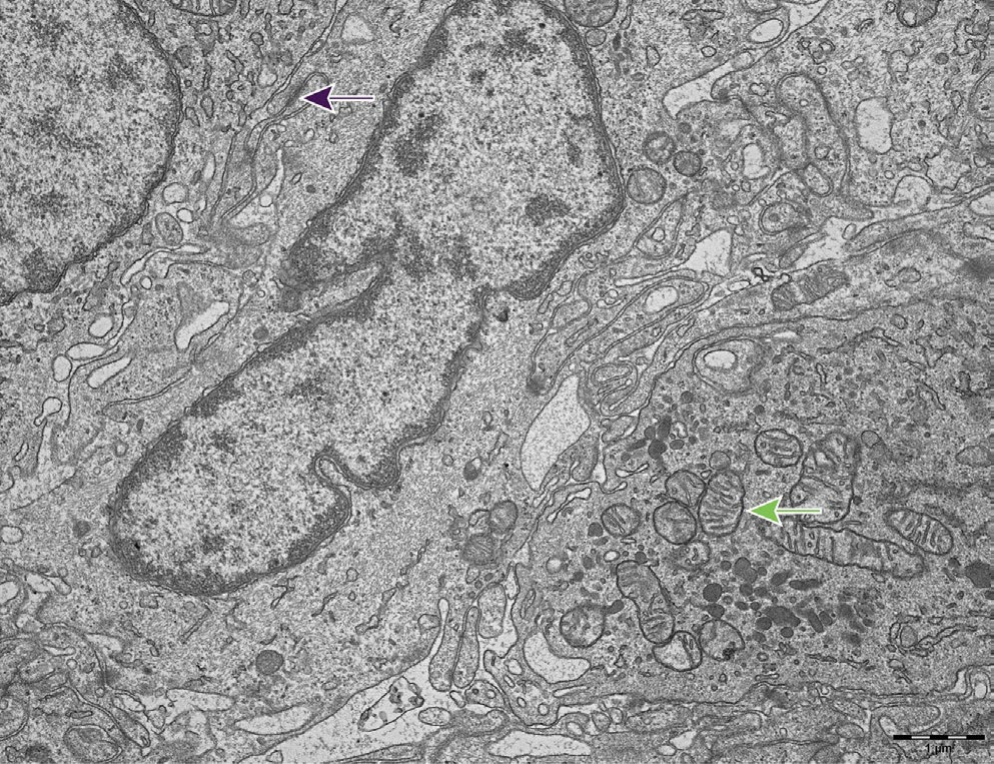
Meningothelial meningioma. Elongated meningioma cells with numerous cytoplasmatic processes with interdigitations that are connected by intercellular junctions, such as desmosomes (purple arrow). Prolonged nuclei with condensed heterochromatin beneath the nuclear membrane. Several organelles, including mitochondria (green arrow), are visualized. No secretory granules are observed. Original magnification ×15,000.

One WHO grade 1 meningothelial meningioma was examined with TEM (Figure 5). Typical meningioma cells with oval nuclei and numerous interdigitations were visualized. The nuclei contained typical condensed heterochromatin. Several desmosomes and different types of organelles, including mitochondria, were observed. No apparent secretory granules were observed. The tissue specimen had a positive expression of NSE, CD56, chromogranin B, and chromogranin C following immunohistochemical procedures. There was no immunoreactivity for chromogranin A, synaptophysin, and secretagogin.

## 4 Discussion

In the present study, we found that 91% of meningiomas expressed NSE, 44% expressed CD56, and 16% expressed chromogranin B. Few tumors expressed chromogranin C, secretagogin, and synaptophysin. None expressed chromogranin A. NSE immunoreactivity was associated with tumor subtype and was upregulated in WHO grade 2 tumors. The immunoreactivity of chromogranin B and NSE was correlated to SSTR2 immunoreactivity. One meningothelial meningioma was investigated using TEM, and no secretory granules were observed.

### 4.1 Neuroendocrine Differentiation Does Not Seem to be Present Human Meningiomas

Chromogranin A and synaptophysin are currently considered the most sensitive and specific among neuroendocrine markers [29, 30]. To our knowledge, chromogranin A has previously not been studied in a larger series of meningiomas. One study did not detect synaptophysin in a series of 15 canine meningiomas [36]. Secretagogin is reported in several CNS tumors, including three investigated meningiomas [24]. In our study, none of the meningiomas expressed chromogranin A, only five meningiomas expressed synaptophysin and two meningiomas expressed secretagogin. These findings suggest that neuroendocrine differentiation in human meningiomas is not present or very rare. We did not detect secretory granules in the single tumor specimen investigated with TEM. Although an increased number of tumors investigated with TEM would have strengthened this finding, studies have demonstrated a correlation between the immunoreactivity of chromogranin and the quantity of secretory granules visualized using TEM [30]. Hence, one may assume that none of the investigated tumors are expected to have secretory granules. The suggested association of MEN1 syndrome and meningiomas may be caused by other inherent factors for patients with MEN1. Examples of this may include hyperprolactinemia from pituitary neuroendocrine tumors (PitNETs), as one review study suggests that prolactin may be involved in pregnancy‐related meningiomas [37], although there is currently no strong evidence for such relations. More importantly, radiotherapy could be used to treat PitNETs, potentially causing radiation‐induced meningiomas [38]. Furthermore, neither the 2021 WHO classification nor the cIMPACT‐NOW updates mention *MEN1* mutations as essential for meningiomas development or diagnostics [1, 39].

Insulinoma‐associated protein 1 (INSM1) has also been suggested as a sensitive and specific neuroendocrine marker. Yet, empirical experience from our hospital (St. Olavs Hospital, Trondheim University Hospital, Norway) has suggested INSM1 to be less sensitive for neuroendocrine tumors in the gastrointestinal tract than chromogranin A and synaptophysin. This has also been confirmed by one study on gastrointestinal neuroendocrine neoplasms, although this study also found INSM1 to be more specific than the traditional neuroendocrine markers [40]. Still, we did not include INSM1 as a neuroendocrine marker.

### 4.2 NSE, CD56, and Chromogranin B Are Not Sufficient Evidence for Neuroendocrine Differentiation

Our findings on NSE and CD56 immunoreactivity in meningiomas correspond with previous research studies, although neuroendocrine proteins have mostly been investigated in smaller studies and case reports [17, 18, 19, 20, 21, 22, 23, 24, 25]. Our study detected NSE in most meningiomas, proving high sensitivity for meningiomas. Two other research papers found variable immunoreactivity of NSE in canine meningiomas, while another paper detected NSE in about 25% of the investigated human meningiomas [25, 36, 41]. NSE may also be detected in tumors without neuroendocrine origin, such as undifferentiated carcinomas or adenocarcinomas, questioning the specificity of NSE as a neuroendocrine marker [29]. In our study, the immunoreactivity of NSE was significantly higher in WHO grade 2 tumors and in some tumor subtypes. Neuroendocrine transdifferentiation is also observed in treatment‐resistant prostate cancer that could be partly due to epigenetic regulations [42]. Higher grade meningiomas are associated with loss of H3K27me3 and this may perhaps also offer some explanation to the observed higher NSE expression in WHO grade 2 meningiomas [39]. NSE was significantly related to SSTR2 immunoreactivity and, similar to NSE, our previous study also found SSTR2 to be upregulated in WHO grade 2 meningiomas [9]. Our study detected CD56 in several meningiomas. CD56 is essential for migration, cell‐binding and differentiation, and also involved in several contact‐mediated interactions between neurons, oligodendrocytes and astrocytes [29, 43]. The marker is also expressed in other brain tumors, including gliomas and medulloblastomas [43, 44]. The presence of NSE and CD56 is not sufficient evidence for neuroendocrine differentiation, as both can be detected in tumors without neuroendocrine origin [29, 43, 44]. Lastly, chromogranin B was detected with weak cytoplasmatic immunoreactivity in 16% of the meningiomas, where some cells had darker granules. The monoclonal antibodies used for chromogranin B and chromogranin C were incubated over two nights. Longer incubation time may indicate lower antibody affinity, increased risk for background staining or false positive staining [45, 46]. Hence, our results for chromogranin B and chromogranin C should be interpreted with care. Overall, the immunoreactivity of NSE, CD56 and chromogranin B in some meningiomas is not sufficient evidence for neuroendocrine differentiation.

### 4.3 Manual and Digital Evaluation of Immunoreactivity Showed Excellent Agreement

The two methods for evaluation of immunoreactivity were significantly correlated, thus providing strong convergent validation of our results. Visual scoring is often considered the gold standard for immunohistochemistry, though it may impose troublesome reproducibility and interrater discordance [35, 47, 48]. As the pixel analyses do not utilize artificial intelligence, it may be vulnerable to artifacts or insufficient image resolution compared to manual scoring. Our tissue specimens contained few artifacts, and the image resolution was high.

### 4.4 Strengths and Limitations of the Study

The large number of patients with meningioma included in our study strengthens our findings. As for limitations, the meningiomas were classified according to the 2016 WHO classification. The recent 2021 WHO classification grades meningiomas with loss of *CDKN2A* and/or *CDKN2B,* or *TERT* promotor mutation as WHO grade 3 tumors [49]. Reclassification of our study material may increase the number of WHO grade 3 tumors. Still, these mutations are rare and would presumably not affect our findings significantly [50]. The use of TMA cores allows for analyzation of large cohorts of tissue samples with a standardized protocol for all included samples. Although there may be concerns regarding tissue representativity, some studies report that TMAs provide similar results to those of whole‐tissue sections [51, 52]. During the TMA preparation, whole‐tissue sections were carefully reviewed by an experienced neuropathologist to ensure the inclusion of representative tissue.

## 5 Conclusion

Our study did not find sufficient evidence to confirm the presence of neuroendocrine differentiation in human meningiomas. Chromogranin B, CD56, and NSE were expressed in some human meningiomas, while the more sensitive and specific neuroendocrine markers chromogranin A and synaptophysin were mostly not detected. NSE had high sensitivity for meningiomas and demonstrated significant associations to higher WHO grade, SSTR2 immunoreactivity, and tumor subtype. Future studies may further investigate *MEN1* status in sporadic meningiomas and its possible relevance for patient outcomes.

## Funding

This work was supported by the Norwegian University of Science and Technology (NTNU) (funding number: 70442762).

## Ethics Statement

This study was conducted according to the principles of the Declaration of Helsinki and has been approved by the Regional Committees for Medical and Health Research Ethics with project number 4.2006.947.

## Conflicts of Interest

The authors declare no conflicts of interest.

## Supporting information


**Table S1:** Primary antibodies for all seven neuroendocrine proteins.
**Table S2:** Spearman's rank‐order correlation between digital score (DS) and staining index (SI) for all seven neuroendocrine proteins.
**Table S3:** Number of positive tumors for each neuroendocrine protein. Tumors with staining index (SI) > 3 were considered as positive tumors. Percentages of positive tumors are given in parenthesis.
**Table S4:** Immunohistochemical results with median score and range for WHO grade 1, WHO grade 2 and collectively for all tumor cases. The TMA cores were evaluated with digital score (DS) and staining index (SI).

## Data Availability

The data that support the findings of this study are available from the corresponding author upon reasonable request.

## References

[1] F. Sahm , P. K. Brastianos , E. B. Claus , et al., “Chapter 7: Meningioma,” in Central Nervous System Tumours, 5th ed., ed. D. N. Louis (International Agency for Research on Cancer, 2021), 283–299.

[2] J. Y. Tsang and G. M. Tse , “Breast Cancer With Neuroendocrine Differentiation: An Update Based on the Latest WHO Classification,” Modern Pathology 34, no. 6 (2021): 1062–1073. 33531618 10.1038/s41379-021-00736-7

[3] S. W. Fine , “Neuroendocrine Tumors of the Prostate,” Modern Pathology 31, no. 1 (2018): 122–132. 29297494 10.1038/modpathol.2017.164

[4] C. D. C. Kamilaris and C. A. Stratakis , “Multiple Endocrine Neoplasia Type 1 (MEN1): An Update and the Significance of Early Genetic and Clinical Diagnosis,” Front Endocrinology (Lausanne) 10 (2019): 339. 10.3389/fendo.2019.00339 PMC6584804 31263451

[5] R. A. Buerki , C. M. Horbinski , T. Kruser , P. M. Horowitz , C. D. James , and R. V. Lukas , “An Overview of Meningiomas,” Future Oncology 14, no. 21 (2018): 2161–2177. 30084265 10.2217/fon-2018-0006 PMC6123887

[6] S. G. Waguespack , “Beyond the “3 Ps”: A Critical Appraisal of the Non‐Endocrine Manifestations of Multiple Endocrine Neoplasia Type 1,” Front Endocrinology (Lausanne) 13 (2022): 1029041. 10.3389/fendo.2022.1029041 PMC9618614 36325452

[7] T. Graillon , P. Romanet , C. Camilla , et al., “A Cohort Study of CNS Tumors in Multiple Endocrine Neoplasia Type 1,” Clinical Cancer Research 30, no. 13 (2024): 2835–2845. 38630553 10.1158/1078-0432.CCR-23-3308

[8] B. Asgharian , Y. J. Chen , N. J. Patronas , et al., “Meningiomas May Be a Component Tumor of Multiple Endocrine Neoplasia Type 1,” Clinical Cancer Research 10, no. 3 (2004): 869–880. 14871962 10.1158/1078-0432.ccr-0938-3

[9] S. E. Tollefsen , A. H. Jarmund , B. Ytterhus , Ø. Salvesen , P. Mjønes , and S. H. Torp , “Somatostatin Receptors in Human Meningiomas—Clinicopathological Aspects,” Cancers 13, no. 22 (2021): 5704. 34830858 10.3390/cancers13225704 PMC8616360

[10] G. Klöppel , “Neuroendocrine Neoplasms: Dichotomy, Origin and Classifications,” Visceral Medicine 33, no. 5 (2017): 324–330. 29177160 10.1159/000481390 PMC5697503

[11] M. Volante , M. P. Brizzi , A. Faggiano , et al., “Somatostatin Receptor Type 2A Immunohistochemistry in Neuroendocrine Tumors: A Proposal of Scoring System Correlated With Somatostatin Receptor Scintigraphy,” Modern Pathology 20, no. 11 (2007): 1172–1182. 17873898 10.1038/modpathol.3800954

[12] A. K. Nazar and S. Basu , “Radiolabeled Somatostatin Analogs for Cancer Imaging,” Seminars in Nuclear Medicine 54, no. 6 (2024): 914–940. 39122608 10.1053/j.semnuclmed.2024.07.001

[13] S. Priyadarshini , D. B. Allison , and A. Chauhan , “Comprehensive Assessment of Somatostatin Receptors in Various Neoplasms: A Systematic Review,” Pharmaceutics 14, no. 7 (2022): 1394. 35890290 10.3390/pharmaceutics14071394 PMC9325105

[14] S. E. Tollefsen , O. Solheim , P. Mjønes , and S. H. Torp , “Meningiomas and Somatostatin Analogs: A Systematic Scoping Review on Current Insights and Future Perspectives,” International Journal of Molecular Sciences 24, no. 5 (2023): 4793. 36902224 10.3390/ijms24054793 PMC10003463

[15] T. A. Hope , M. Pavel , and E. K. Bergsland , “Neuroendocrine Tumors and Peptide Receptor Radionuclide Therapy: When Is the Right Time?,” Journal of Clinical Oncology 40, no. 24 (2022): 2818–2829. 35649195 10.1200/JCO.22.00176 PMC9390818

[16] F. Sahm , L. Bertero , S. Brandner , et al., “European Association of Neuro‐Oncology Guideline on Molecular Testing of Meningiomas for Targeted Therapy Selection,” Neuro‐Oncology 27, no. 4 (2025): 869–883. 39577862 10.1093/neuonc/noae253 PMC12083233

[17] M. Hu , Y. Tang , G. Long , D. Zhang , J. L. Kresak , and J. Lai , “Primary Extracranial Meningioma of Mastoid in a Patient With History of Skin Squamous Cell Carcinoma, Lung Adenocarcinoma and Prostatic Carcinoma,” Anticancer Research 39, no. 6 (2019): 3197–3201. 31177167 10.21873/anticanres.13458

[18] T. Hayashi , R. Haba , Y. Kushida , et al., “Cytopathologic Features of Orbital Intraosseous Chordoid Meningioma: Report of a Case and Distinction From Other Myxoid/Mucoid Tumors,” Diagnostic Cytopathology 38, no. 11 (2010): 818–821. 20091894 10.1002/dc.21326

[19] M. H. Sanei , N. Berjis , P. Mahzouni , and A. Naimi , “A Case of Neck Ectopic Meningioma,” Neuropathology 28, no. 2 (2008): 157–159. 18179413 10.1111/j.1440-1789.2007.00835.x

[20] H. Takei , A. Rivera , H. Suzuki , A. Bahrami , and S. Z. Powell , “Jugular Foramen Chordoid Meningioma,” Pathology International 56, no. 7 (2006): 397–401. 16792549 10.1111/j.1440-1827.2006.01976.x

[21] M. Payano , Y. Kondo , K. Kashima , et al., “Two Cases of Nondura‐Based Clear Cell Meningioma of the Cauda Equina,” APMIS 112, no. 2 (2004): 141–147. 15056231 10.1111/j.1600-0463.2004.apm1120209.x

[22] S. Al‐Sarraj , A. King , A. J. Martin , J. Jarosz , and P. L. Lantos , “Ultrastructural Examination Is Essential for Diagnosis of Papillary Meningioma,” Histopathology 38, no. 4 (2001): 318–324. 11318897 10.1046/j.1365-2559.2001.01128.x

[23] M. L. Tena‐Suck , M. A. Collado‐Ortìz , C. Salinas‐Lara , R. García‐López , N. Gelista , and D. Rembao‐Bojorquez , “Chordoid Meningioma: A Report of Ten Cases,” Journal of Neuro‐Oncology 99, no. 1 (2010): 41–48. 20094774 10.1007/s11060-009-0097-9

[24] I. Pipp , L. Wagner , K. Rössler , H. Budka , and M. Preusser , “Secretagogin Expression in Tumours of the Human Brain and Its Coverings,” APMIS 115, no. 4 (2007): 319–326. 17504298 10.1111/j.1600-0463.2007.apm_590.x

[25] A. Artlich and D. Schmidt , “Immunohistochemical Profile of Meningiomas and Their Histological Subtypes,” Human Pathology 21, no. 8 (1990): 843–849. 1696924 10.1016/0046-8177(90)90054-9

[26] F. Pecori Giraldi , M. R. Terreni , C. Andreotti , et al., “Meningioma Presenting With Cushing's Syndrome: An Unusual Clinical Presentation,” Annals of Neurology 53, no. 1 (2003): 138–142. 12509860 10.1002/ana.10454

[27] D. Klimstra , G. Klöppel , S. La Rosa , and G. Rindi , “Classification of Neuroendocrine Neoplasms of the Digestive System,” in WHO Classification of Tumours Editorial Board Digestive System Tumours [Internet], 5th ed., ed. M. Washington (International Agency for Research on Cancer, 2019), https://publications.iarc.fr/579.

[28] A. M. Bellizzi , “Immunohistochemistry in the Diagnosis and Classification of Neuroendocrine Neoplasms: What Can Brown Do for You?,” Human Pathology 96 (2020): 8–33. 31857137 10.1016/j.humpath.2019.12.002 PMC7177196

[29] P. Mjønes , L. Sagatun , I. S. Nordrum , and H. L. Waldum , “Neuron‐Specific Enolase as an Immunohistochemical Marker Is Better Than Its Reputation,” Journal of Histochemistry and Cytochemistry 65, no. 12 (2017): 687–703. 28972818 10.1369/0022155417733676 PMC5714096

[30] R. Bhargava , “Chapter 8. Immunohistology of Carcinoma of Unknown Primary Site: Neuroendocrine Antibodies,” in Diagnostic Immunohistochemistry: Theranostics and Genomic Applications, 5th ed., ed. D. Dabbs (Elsevier, 2019), 238–239.

[31] A. Perry , D. N. Louis , H. Budka , et al., “Meningiomas,” in WHO Classification of Tumours of the Central Nervous System, 4th ed. (IARC, 2016), 231–245.

[32] M. B. Arnli , T. L. Winther , S. Lydersen , and S. H. Torp , “Prognostic Value of ErbB2/HER2 in Human Meningiomas,” PLoS One 13, no. 10 (2018): e0205846. 30335819 10.1371/journal.pone.0205846 PMC6193666

[33] T. Backer‐Grøndahl , B. H. Moen , and S. H. Torp , “The Histopathological Spectrum of Human Meningiomas,” International Journal of Clinical and Experimental Pathology 5, no. 3 (2012): 231–242. 22558478 PMC3341686

[34] M. B. Arnli , T. Backer‐Grøndahl , B. Ytterhus , et al., “Expression and Clinical Value of EGFR in Human Meningiomas,” PeerJ 5 (2017): e3140. 28367377 10.7717/peerj.3140 PMC5374971

[35] F. Varghese , A. B. Bukhari , R. Malhotra , and A. De , “IHC Profiler: An Open Source Plugin for the Quantitative Evaluation and Automated Scoring of Immunohistochemistry Images of Human Tissue Samples,” PLoS One 9, no. 5 (2014): e96801. 24802416 10.1371/journal.pone.0096801 PMC4011881

[36] K. F. Barnhart , J. Wojcieszyn , and R. W. Storts , “Immunohistochemical Staining Patterns of Canine Meningiomas and Correlation With Published Immunophenotypes,” Veterinary Pathology 39, no. 3 (2002): 311–321. 12014495 10.1354/vp.39-3-311

[37] Y. Laviv , V. Ohla , and E. M. Kasper , “Unique Features of Pregnancy‐Related Meningiomas: Lessons Learned From 148 Reported Cases and Theoretical Implications of a Prolactin Modulated Pathogenesis,” Neurosurgical Review 41, no. 1 (2018): 95–108. 27312026 10.1007/s10143-016-0762-3

[38] C. S. Gillespie , A. I. Islim , B. A. Taweel , et al., “The Growth Rate and Clinical Outcomes of Radiation Induced Meningioma Undergoing Treatment or Active Monitoring,” Journal of Neuro‐Oncology 153, no. 2 (2021): 239–249. 33886110 10.1007/s11060-021-03761-3 PMC8211577

[39] F. Sahm , K. D. Aldape , P. K. Brastianos , et al., “cIMPACT‐NOW Update 8: Clarifications on Molecular Risk Parameters and Recommendations for WHO Grading of Meningiomas,” Neuro‐Oncology 27, no. 2 (2024): noae170. 10.1093/neuonc/noae170 PMC11812049 39212325

[40] K. E. McHugh , S. Mukhopadhyay , E. E. Doxtader , C. Lanigan , and D. S. Allende , “INSM1 Is a Highly Specific Marker of Neuroendocrine Differentiation in Primary Neoplasms of the Gastrointestinal Tract, Appendix, and Pancreas,” American Journal of Clinical Pathology 153, no. 6 (2020): 811–820. 32128564 10.1093/ajcp/aqaa014

[41] P. Montoliu , S. Añor , E. Vidal , and M. Pumarola , “Histological and Immunohistochemical Study of 30 Cases of Canine Meningioma,” Journal of Comparative Pathology 135, no. 4 (2006): 200–207. 17049358 10.1016/j.jcpa.2006.06.006

[42] J. Jiang , D. Han , J. Wang , W. Wen , R. Zhang , and W. Qin , “Neuroendocrine Transdifferentiation in Human Cancer: Molecular Mechanisms and Therapeutic Targets,” MedComm 5, no. 10 (2024): e761. 39372390 10.1002/mco2.761 PMC11450264

[43] E. P. Weledji and J. C. Assob , “The Ubiquitous Neural Cell Adhesion Molecule (N‐CAM),” Annals of Medicine and Surgery 3, no. 3 (2014): 77–81. 25568792 10.1016/j.amsu.2014.06.014 PMC4284440

[44] P. Marques , S. Barry , E. Carlsen , et al., “The Expression of Neural Cell Adhesion Molecule and the Microenvironment of Pituitary Neuroendocrine Tumours,” Journal of Neuroendocrinology 33, no. 12 (2021): e13052. 34708902 10.1111/jne.13052

[45] J. A. Ramos‐Vara , “Technical Aspects of Immunohistochemistry,” Veterinary Pathology 42, no. 4 (2005): 405–426. 16006601 10.1354/vp.42-4-405

[46] S. Magaki , S. A. Hojat , B. Wei , A. So , and W. H. Yong , “An Introduction to the Performance of Immunohistochemistry,” Methods in Molecular Biology 1897 (2019): 289–298. 30539453 10.1007/978-1-4939-8935-5_25 PMC6749998

[47] A. L. Hein , M. Mukherjee , G. A. Talmon , et al., “QuPath Digital Immunohistochemical Analysis of Placental Tissue,” Journal of Pathology Informatics 12 (2021): 40. 34881095 10.4103/jpi.jpi_11_21 PMC8609285

[48] P. W. Hamilton , P. Bankhead , Y. Wang , et al., “Digital Pathology and Image Analysis in Tissue Biomarker Research,” Methods 70, no. 1 (2014): 59–73. 25034370 10.1016/j.ymeth.2014.06.015

[49] D. N. Louis , A. Perry , P. Wesseling , et al., “The 2021 WHO Classification of Tumors of the Central Nervous System: A Summary,” Neuro‐Oncology 23, no. 8 (2021): 1231–1251. 34185076 10.1093/neuonc/noab106 PMC8328013

[50] C. Birzu , M. Peyre , and F. Sahm , “Molecular Alterations in Meningioma: Prognostic and Therapeutic Perspectives,” Current Opinion in Oncology 32, no. 6 (2020): 613–622. 32890025 10.1097/CCO.0000000000000687

[51] E. Fernebro , M. Dictor , P. O. Bendahl , M. Fernö , and M. Nilbert , “Evaluation of the Tissue Microarray Technique for Immunohistochemical Analysis in Rectal Cancer,” Archives of Pathology & Laboratory Medicine 126, no. 6 (2002): 702–705. 12033959 10.5858/2002-126-0702-EOTTMT

[52] A. Hoos , M. J. Urist , A. Stojadinovic , et al., “Validation of Tissue Microarrays for Immunohistochemical Profiling of Cancer Specimens Using the Example of Human Fibroblastic Tumors,” American Journal of Pathology 158, no. 4 (2001): 1245–1251. 11290542 10.1016/S0002-9440(10)64075-8 PMC1891917

