# Supplementary material for: Human Meningiomas Reveal No Evidence of Neuroendocrine Differentiation
Source: APMIS. 2026 Mar 2;134(3):e70177. doi: 10.1111/apm.70177 (PMC12951546; doi:10.1111/apm.70177)
Supplement: Supplementary file 1 — Table S1: Primary antibodies for all seven neuroendocrine proteins. Table S2: Spearman's rank‐order correlation between digital score (DS) and staining index (SI) for all seven neuroendocrine proteins. Table S3: Number of positive tumors for each neuroendocrine protein. Tumors with staining index (SI) > 3 were considered as positive tumors. Percentages of positive tumors are given in parenthesis. Table S4: Immunohistochemical results with median score and range for WHO grade 1, WHO grade 2 and collectively for all tumor cases. The TMA cores were evaluated with digital score (DS) and staining index (SI). [file APM-134-0-s001.docx]

**SUPPLEMENTARY INFORMATION**

**Article title:** Human meningiomas reveal no evidence of neuroendocrine differentiation

**Journal name:** Journal of Pathology, Microbiology and Immunology - the APMIS journal

**Authors:** Sofie Eline Tollefsen^*^, Anders Hagen Jarmund, Ole Solheim, Ida Kaalhus Nordahl, Thi My Linh Hoang, Anette H. Skjervold, Patricia Mjønes, Sverre Helge Torp

***Correspondence:** Sofie Eline Tollefsen, MD PhD, Department of Clinical and Molecular Medicine, Faculty of Medicine and Health Sciences, Norwegian University of Science and Technology, 7491 Trondheim, Norway; E-mail: [sofie.e.tollefsen@ntnu.no](mailto:sofie.e.tollefsen@ntnu.no). Orcid-ID: [0000-0002-9521-1032](https://orcid.org/0000-0002-9521-1032).

**Supplementary material 1**

**Table S1.** Primary antibodies for all seven neuroendocrine proteins.

| CD56 | MRQ-42, monoclonal (rabbit), pH 9, 1:500 dilution, 40 minutes incubation in room temperature, CD56, cat#MRQ-42, Cell Marque, Sigma-Aldrich |
| --- | --- |
| Chromogranin A | Chromogranin A clone DAK-A3, monoclonal (mouse), pH 9, 1:100 dilution, 40 minutes incubation in room temperature, chromogranin A, cat#M0869, DAKO |
| Chromogranin B | Chromogranin B clone 914334, monoclonal (mouse), pH 9, 1:250 dilution, two nights incubation in 4°C, chromogranin B, cat#MAB8868, R&D Systems |
| Chromogranin C | Chromogranin C clone EPR23876-52, monoclonal (rabbit), pH 9, 1:2000 dilution, two nights incubation in 4°C, chromogranin C, cat#ab275028, Abcam |
| Neuron-Specific Enolase | Neuron-Specific Enolase clone BBS/NC/VI-H14, monoclonal (mouse), pH 9, 1:200 dilution, 40 minutes incubation in room temperature, NSE, cat#IR612, DAKO |
| Secretagogin | Secretagogin clone 778518, monoclonal (mouse), pH 9, 1:1000 dilution, overnight incubation in 4°C, secretagogin, cat#MAB4878, R&D Systems |
| Synaptophysin | Synaptophysin clone 27G12, monoclonal (mouse), pH 9, 1:50 dilution, 40 minutes incubation in room temperature, synaptophysin, cat#NCL-L-SYNAP-299, Leica Biosystems |

**Supplementary material 2**

**Table S2.** Spearman’s rank-order correlation between digital score (DS) and staining index (SI) for all seven neuroendocrine proteins.

|  | **Digital score versus Staining index** |
| --- | --- |
| CD56 | r = .915  *p* < .001 |
| Chromogranin A | N/A* |
| Chromogranin B | r = .626  *p* < .001 |
| Chromogranin C | r = .599  *p <* .001 |
| NSE | r = .745  *p <* .001 |
| Secretagogin | r = .401  *p <* .001 |
| Synaptophysin | r = .165  *p* = .036 |

Abbreviations: Neuron-specific enolase (NSE); Neutral cell adhesion molecule (CD56). *Not applicable (N/A): SI ranges from 0-0, not possible to calculate correlation based on these values.

**Supplementary material 3**

**Table S3.** Number of positive tumors for each neuroendocrine protein. Tumors with staining index (SI) > 3 were considered as positive tumors. Percentages of positive tumors are given in parenthesis.

|  | **CD56** | | **Chromogranin A** | **Chromogranin B** | **Chromogranin C** | **NSE** | **Secretagogin** | **Synaptophysin** |
| --- | --- | --- | --- | --- | --- | --- | --- | --- |
| **Positive tumors (%)** | | 70 (44%) | 0 (0%) | 26 (16.3%) | 12 (7.5%) | 144 (91.1%) | 2 (1.2%) | 5 (3.1%) |
| **Excluded cases** | 3 | | 1 | 2 | 1 | 4 | 1 | 1 |

Abbreviations: Neuron-specific enolase (NSE); Neutral cell adhesion molecule (CD56).

**Supplementary material 4**

**Table S4.** Immunohistochemical results with median score and range for WHO grade 1, WHO grade 2 and collectively for all tumor cases. The TMA cores were evaluated with digital score (DS) and staining index (SI).

|  | **Digital Score (DS)**  **Median [range]** | | | **Staining index (SI)**  **Median [range]** | | | **Excluded cases** |
| --- | --- | --- | --- | --- | --- | --- | --- |
|  | **WHO grade 1** | **WHO grade 2** | **All cases** | **WHO grade 1** | **WHO grade 2** | **All cases** |  |
| CD56 | 1.46  [1.1-2.9] | 1.41  [1.2-3.2] | 1.46  [1.1-3.2] | 2  [0-9] | 2  [0-9] | 2  [0-9] | 3 |
| Chromogranin A | 1.01  [1.0-1.1] | 1.01  [1.0-1.1] | 1.01  [1.0-1.1] | 0  [0-0] | 0  [0-0] | 0  [0-0] | 1 |
| Chromogranin B | 1.16  [1.0-1.7] | 1.15  [1.0-2.0] | 1.15  [1.0-2.0] | 1  [0-4] | 1  [0-4] | 1  [0-4] | 2 |
| Chromogranin C | 1.04  [1.0-1.5] | 1.05  [1.0-1.2] | 1.04  [1.0-1.5] | 2  [0-4] | 2  [0-4] | 2  [0-4] | 1 |
| NSE | 2.30  [1.2-3.8] | 2.40  [1.3-3.5] | 2.34  [1.2-3.8] | 9  [1-9] | 9  [2-9] | 9  [1-9] | 4 |
| Secretagogin | 1.03  [1.0-2.2] | 1.02  [1.0-1.4] | 1.02  [1.0-2.2] | 0  [0-6] | 0  [0-2] | 0  [0-6] | 1 |
| Synaptophysin | 1.01  [1.0-1.2] | 1.01  [1.0-1.1] | 1.01  [1.0-1.2] | 1  [0-4] | 1  [0-4] | 1  [0-4] | 1 |

Abbreviations: Neuron-specific enolase (NSE); Neutral cell adhesion molecule (CD56)
